# How do introductory field biology students feel? Journal reflections provide insight into student affect

**DOI:** 10.1002/ece3.9454

**Published:** 2022-11-15

**Authors:** Kira A. Treibergs, David Esparza, Jeannie A. Yamazaki, Marc Goebel, Michelle K. Smith

**Affiliations:** ^1^ Department of Natural Resources and the Environment Cornell University Ithaca New York USA; ^2^ Department of Ecology and Evolutionary Biology Cornell University Ithaca New York USA

**Keywords:** achievement emotions, affect, connection to nature, field course, identity, motivation, prosocial opportunities, undergraduate

## Abstract

An understanding of both cognitive and affective domains of learning is critical to promoting undergraduate student success in biology. Field courses—which support student learning, observation, and experimentation in the outdoors—have been shown to be effective in supporting cognitive student outcomes. However, less is known about students' affective responses during field instruction. To better understand the affective domain in this course type, we asked students enrolled in a campus‐based introductory field biology course to engage in weekly reflective journaling over the course of a semester. We employed inductive and deductive coding of over 700 field journal reflections using the Model of the Affective Domain for the Geosciences as a conceptual basis. Informed by our results, we present a theoretically‐driven, five‐part Framework of Student Affect in Field Biology and in‐depth and novel insights into what students feel, believe, and value as they participate in an undergraduate field course. Our framework and coding results can be used by field course instructors to understand how to better design experiences that leave students feeling confident in their abilities, interested to learn more about nature, and empowered to persist in the discipline.

## Graphical abstract

To better understand the affective domain of students participating in field biology, we coded over 700 journal reflections and developed a Framework of Student Affect in Field Biology. We share novel insights into student affect and make practical recommendations for instructors of undergraduate field biology courses.

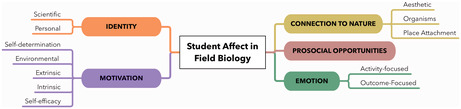

## 1 INTRODUCTION

The affective domain of learning refers to the attitudes, emotions, feelings, and values a student experiences during the learning process, which can positively or negatively impact what students learn (Hart, 1989; Vermunt, 1996). In biology education, instructors have typically focused on assessing students' knowledge of biology concepts throughout their participation in coursework, with less consideration of how biology instruction impacts students in the affective domain (Shinbrot et al., 2022; Trujillo & Tanner, 2014). However, a large and growing body of research suggests that the affective domain and students' academic achievement are inextricably linked (Thompson & Mintzes, 2002). Given the importance of the affective domain, several studies have worked to characterize student affect in biology lectures and laboratory courses (Ballen et al., 2017; Cleveland et al., 2017; Cooper et al., 2020; Olimpo et al., 2016; Starr et al., 2020). Yet, fewer studies have worked to understand the affective domain of learning as it exists in undergraduate field courses in the life sciences.

Undergraduate field courses—which support student‐centered engagement in outdoor instruction, experimentation, sampling, and observation—are a common method of providing students with fieldwork experiences. A unifying goal of field courses is to facilitate experiential learning, a process by which students build disciplinary knowledge through engagement in concrete experiences (Kolb, 2015). Field courses promote experiential learning by asking students to directly engage in the practices of field scientists (e.g., observation and hands‐on sample collection) to explain biological phenomena. By adopting a “learning‐by‐doing” approach, field courses can help students contextualize the science content they have learned in the classroom to aid in their understanding of complex natural and environmental processes (Klemow et al., 2019). They also improve upon students' achievement and retention in biology degree programs (Beltran et al., 2020).

By providing engaging and immersive experiences outside of the classroom, field courses support student development in the affective domain (O’Connell et al., 2021, 2022). Recent studies have indicated that field instruction can improve science identity (Race et al., 2021), science self‐efficacy (Beltran et al., 2020), and motivation in the discipline (Peasland et al., 2021). In addition, field courses support affective student outcomes such as place attachment (Semken & Freeman, 2008), an increased appreciation for the sociocultural history of outdoor spaces (Alagona & Simon, 2010), and pro‐environmental attitudes (Jolley et al., 2018).

Despite these outcomes, field courses face an uncertain future in the undergraduate science curriculum. They require intensive planning, organization, and a unique level of adaptability from instructors who may lack support from their institutions or experience in facilitating such courses (Fleischner et al., 2017). Additionally, students occasionally find field experiences to be frustrating (Baum et al., 2012), anxiety‐inducing (Cotton, 2009), or even boring (Boyle et al., 2007). Students who identify with minoritized races/ethnicities and other identities can sometimes feel uncomfortable, a lack of belonging, or even unsafe in outdoor learning environments (Demery & Pipkin, 2021; Malm et al., 2020; O’Brien et al., 2020). Further, field courses can pose a wide range of accessibility, mobility, and safety challenges that can impede or even bar students with disabilities from participating (Atchison et al., 2019; Kingsbury et al., 2020). In consideration of these challenges, the National Academies of Science, Engineering, and Medicine (National Research Council, 2012) called for future research on the learning outcomes best achieved through field instruction and further research into the affective domain of field learning.

One method to capture and better understand students' affective insights is reflective journaling (Hubbs & Brand, 2010; Tammu, 2022). During reflective journaling, students revisit their course experiences to produce a written narrative that represents their thoughts, emotions, values, and beliefs throughout the learning process (Boud et al., 1985). Reflective journaling can help students make meaning of what they learned through contextualizing new content in relation to their prior knowledge and past experiences (Lew & Schmidt, 2011; Moon, 1999). Furthermore, a study that randomly assigned introductory biology students to reflectively journal or write a scientific report found that students who wrote reflective journals used a wider array of metacognitive strategies when studying for course exams and exhibited increased exam performance (McCrindle & Christensen, 1995).

While reflection is a critical aspect of the experiential learning process (Jordi, 2011), only a handful of studies have used reflective journals to chronicle students' affective responses to field courses. Recently, Race et al. (2021) utilized reflective journal prompts intentionally designed to assess shifts in student science identity, science self‐efficacy, and sense of community. Moreover, Scott et al. (2019) analyzed students' reflections to similarly structured prompts following their participation in a short‐term, residential field program, finding students were more likely to make positive affective statements rather than negative statements about fieldwork. While these existing studies have effectively utilized specific prompts to target certain affective outcomes of fieldwork experiences, no prior study, to our knowledge, has implemented general prompts to capture a broader range of affective outcomes. Through qualitative analysis of students' reflective journaling about their field course, we answer the question: What affective outcomes do students discuss when reflecting on field experiences that are part of an introductory campus‐based field biology course? Informed by the affective responses captured from a semester of students' reflective journaling, we present a Framework of Student Affect in Field Biology.

## 2 CONCEPTUAL MODEL

In this study, we adopt van der Hoeven Kraft et al.'s (2011) Model of the Affective Domain in the Geosciences to provide a conceptual foundation for our qualitative analysis. This model posits that the affective responses students exhibit towards field instruction are linked to the ways students maintain interest in, connect to, or learn from geoscience instruction. This model divides the affective domain into three main constructs: (1) motivation, which concerns a student's willingness to engage in an activity or task; (2) emotion, referring to the “positive and negative” feelings students experience in geoscience courses; and (3) connections with Earth, regarding how people connect with and appreciate natural spaces and geological processes. The model further includes intersections between these three constructs, suggesting that instructors consider how their instruction promotes social interaction (i.e., prosocial opportunities), reaffirms student identities, and strengthens their confidence in their abilities (i.e., self‐efficacy). The authors proposed this model to encourage geoscience education researchers and practitioners to more purposefully consider how to foster these positive affective outcomes.

The Model of the Affective Domain in the Geosciences is well‐suited as an initial conceptual basis to characterize student affect during field biology coursework. Biology and geoscience curricula both emphasize the importance of field courses (Klemow et al., 2019; Mogk & Goodwin, 2012) for students' cognitive and affective development. In our qualitative analysis, we applied this model to characterize field journals completed by students enrolled in a campus‐based field biology course. In applying this model to a new disciplinary context, we drew on additional concepts from a variety of social and psychological theories to expand upon the original model and develop the Framework of Student Affect in Field Biology, which is discussed in more detail below.

## 3 MATERIALS AND METHODS

### 3.1 Contextual information

#### 3.1.1 Participant selection and course description

**FIGURE 1:**
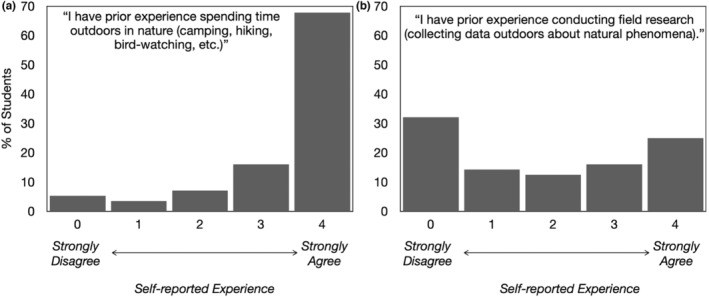
Students self‐reported prior experience, indicated by stating agreement or disagreement with statements related to having (a) prior outdoor experience and (b) prior field research experience. Students (*n* = 56) rated their agreement with each experience statement on a scale of 0 (Strongly Disagree) to 4 (Strongly Agree).

The participants represent a convenience sample of two separate sections of students (*n*_section 1_ = 30, *n*_section 2_ = 31) enrolled in a campus‐based course entitled “Introductory Field Biology” at a northeastern, research‐intensive, doctoral‐granting institution. This introductory field biology course teaches ornithology, forest ecology, entomology, limnology, and herpetology concepts. Students often take this course because it is required for the Environment and Sustainability Science major, an interdisciplinary major that includes a concentration in biology and applied ecology. Many of the students have previous outdoor experiences, and showed a range of experience with field research (Figure 1). Self‐reported student demographic information is described in Table 1.

**TABLE 1:** Combined student demographics in two sections of “Introductory Field Biology”

Category	% Of students (*n* = 61)
Gender identity	
Female	67.2
Male	22.9
Not disclosed^a^	9.8
Race/Ethnicity	
White	47.5
Hispanic/Latiné	16.4
Asian	13.3
Black/African American	6.5
American Indian/Alaska Native	6.5
Not disclosed^a^	9.8
Academic year	
First‐year	4.9
Sophomore	65.6
Junior	26.2
Senior	3.3

^a^ The “Not disclosed” category for each demographic category is comprised of students who did not respond to the demographic survey.

This field biology course was designed to introduce students to fieldwork skills, practice collaboration, and experience authentic self‐directed field research. Course learning outcomes listed that students will be able to: (1) identify and characterize ecosystem types, ecological communities, and habitats in the northeastern region based on key structural features, associated taxa, and the physical environment, (2) identify approximately 200 common taxa of plants and animals in the northeastern region, and understand the natural history of those species and their relationship to the environment, (3) develop an understanding of field research methods and approaches in a variety of ecological disciplines, (4) formulate research questions from field observations, develop a sample design, collect field data, and interpret and discuss their results in relation to research questions, and (5) demonstrate equitable collaboration as they design, plan, execute and communicate the results of a field research project.

**FIGURE 2:**
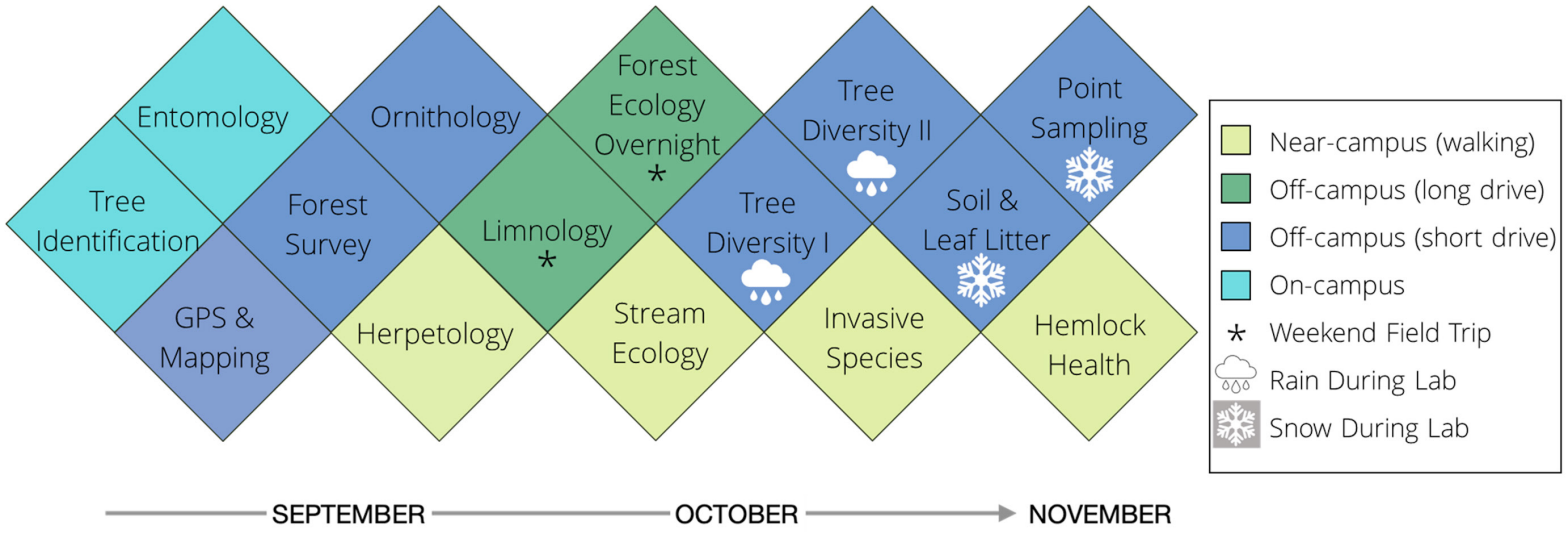
Field lab topics in Introductory Field Biology in 2019. Each box represents an independent field lab associated with the course, colored by location relative to campus. Boxes with asterisks indicate weekend labs that occurred outside of class time. All other experiences occurred during the scheduled time for the course. Boxes with rain or snow icons indicate labs where one or both sections experienced precipitation. From August through November, outdoor temperatures ranged from 26°C through −4°C in November.

In addition to a weekly indoor lecture, students were enrolled in two separate sections that each attended semiweekly 3‐h labs, which utilized a mixture of field‐based and classroom‐based instruction. In total, 13 weekday field labs were held during class time throughout the semester from September to November, along with two weekend experiences (Figure 2), resulting in a total of 15 unique field labs. On occasion, topic area guest lecturers (e.g., faculty, industry experts) were invited to certain field labs to teach students about disciplinary concepts and provide perspectives on careers in these areas.

#### 3.1.2 Field journal assignment

The data used in this study are drawn from course artifacts—student field journals that were written in 2019. Each week, students submitted electronic field journal entries corresponding to each field lab. When presenting the assignment, instructors emphasized the importance of field journaling by describing the value of collecting knowledge for a scientist (e.g., an opportunity to process one's observations, pose questions, and state ideas) and explaining how observations and reflections from historic field journals profoundly influenced a modern understanding of organisms or ecosystems (e.g., providing a historical record of presence or abundance that could inform the conservation of a now‐threatened species) (Canfield, 2011).

Instructors included field journal guidelines that outlined expectations for each section of the journal entry, which students used each week to guide their writing. Graduate teaching assistants used these guidelines to grade entries for completion rather than content. A complete journal entry received 3 points and included descriptions of (a) the field site, (b) weather conditions, (c) lab activities, and (d) species observed, along with (e) a reflective journaling section (hereafter called “field journal reflections”) in response to the field lab experience. Journal entries received 2.5 points if one section was missing, 2 points if two sections were missing, and 1 point if three or more sections were missing. Ultimately, field journals comprised 10% of the final course grade.

Field journal guidelines provided the following directions for the reflective journaling section: “The goal is to reflect on what you did that day so that you will remember as much of the experience as possible. Reflections should be at least one‐half page in length and include: (1) what you thought of the lab, (2) what you found the most fascinating, (3) an explanation of lab challenges/limitations and/or suggestions for an alternative approach, and (4) any interesting things that happened, lab‐related or otherwise.”

We analyzed each entry written by the 61 students who participated in up to 15 different field labs throughout the semester, for a total of 743 field journal reflections. Due to the personal nature of the field journal reflections, students were specifically asked to give consent to the research team for the analysis and dissemination of quotes from their journal entries. This research has been approved by Cornell University's Institutional Review Board under protocol #1708007347.

#### 3.1.3 Author positionality statement

The authors of this paper come from a variety of academic backgrounds including biology education research, environmental science, evolutionary biology, field biology, forest ecology, genetics, and marine ecology.

Three authors were directly affiliated with the “Introductory Field Biology” course being studied: K.T was an Active Learning Initiative postdoctoral scholar who supported the development of evidence‐based teaching innovations in the course from 2019 to 2021; J.Y. was a student in the course in Fall 2018 and undergraduate teaching assistant for the course in Fall 2019; and M.G. has been one of two co‐instructors of the course from 2014 to 2020 and sole course instructor in 2021. The authors D.E. and M.S. were not affiliated with teaching the course. All authors have taken, taught, and are proponents of field‐based instruction. To mitigate biases in our research, we used several different practices to support validity, trustworthiness, and reliability (see Section 3.2.2 below).

### 3.2 Qualitative analysis

#### 3.2.1 Codebook generation and framework development

Prior to coding all field journal reflections, the authors K.T., D.E., and J.Y. created the codebook through four iterative rounds of inductive and deductive coding. For each of these four rounds, each coder independently examined a new subset of 45 randomly selected journal reflections, totaling 180 unique journal reflections (approximately 25% of the total dataset of 743 journal entries) across each of these rounds (Creswell, 2008). Following each round, the coders met to discuss coding and to revise the codebook, ultimately reaching consensus and thematic saturation at the fourth round of analysis.

In the first round of codebook generation, coders derived a priori parent codes (e.g., motivation, emotion) from the Model of the Affective Domain for the Geosciences (van der Hoeven Kraft et al., 2011). In the subsequent rounds of codebook development, the same three coders evaluated entries according to the relevant constructs from the Model of the Affective Domain for Geosciences (van der Hoeven Kraft et al., 2011) and took note of affective sentiments that students commonly expressed in their field journals that were not explicitly captured by the model. For each of these rounds, the coders integrated additional affective theoretical frameworks into the codebook that captured emergent themes (Table 2). These frameworks are discussed in the subsequent paragraphs.

**TABLE 2:** Framework of student affect in Introductory Field Biology: Code and sub‐code descriptions

Code/Sub‐code	Description
Identity (*κ* = .903)^a^	A student connects field experiences to who they are as a person (personal identity) and/or who they are as a scientist (science identity) (Brickhouse et al., 2000; Carlone & Johnson, 2007; Gee, 2000)
Motivation (*κ* = .764)	A student feels an impetus to engage in a behavior during the field experience (Glynn et al., 2011). Motivation can differ based on level (i.e., how motivated one is) and by orientation (i.e., the underlying attitudes and goals behind the motivation) (Ryan & Deci, 2000)
Environmental motivation	A student values or disvalues specific field experiences for their potential for helping to improve or better understand the natural world (Darner, 2009, 2012; Pelletier et al., 1998)
Extrinsic motivation	A student values or disvalues specific field experiences for their utility in supporting their accomplishment of an academic or career goal or some other “separable outcome” (Glynn et al., 2011; Mazlo et al., 2002; Ryan & Deci, 2000)
Intrinsic motivation	A student values or disvalues specific field experiences for engaging their personal interest and/or curiosity or for providing them with inherent satisfaction (Glynn et al., 2011, Ryan & Deci, 2000)
Self‐determination	A student describes independent actions they will or will not take to improve their learning of class concepts outside of class (Deci & Ryan, 1985)
Self‐efficacy	A student describes feeling that they can or cannot do well in learning or engaging with the field experience activities (Glynn et al., 2011; Lawson et al., 2007)
Connection to nature (*κ* = .928)	A student feels unification with, a sense of belonging to, and/or deep appreciation for the natural environment and living organisms (Restall & Conrad, 2015; Wilson, 1984)
Connection to aesthetic	A student mentions appreciating or not appreciating some aspects of the field experience for aesthetic reasons (van der Hoeven Kraft et al., 2011)
Connection to organisms	A student reflects on a positive or negative connection they made to a specific organism(s) through experiencing it in person or expressing a sentiment of either feeling drawn to or repulsed by the organism (Nisbet et al., 2009; Ulrich, 1993)
Place attachment	A student describes a connection between themselves and a place they visited during a field experience (Kudryavtsev et al., 2012; Lewicka, 2011)
Prosocial opportunities (*κ* = .785)	A student reflects on a positive or negative interaction(s) they had with another person or group of people about fieldwork (van der Hoeven Kraft et al., 2011)
Emotion (*κ* = .758)	A student describes a positive or negative emotion they experienced when participating in a field experience (activity‐focused emotions). A student may also describe a positive or negative emotion felt in anticipation of or following the field experience (outcome‐focused emotions) (Pekrun et al., 2011)

^**a**^ Cohen’s Kappa (*κ*), a measure calculated to determine the reliability between the two primary coders K.T. and D.E. is listed for each code. Cohen’s *κ* values ranging from .810–1.000 are considered to be almost perfect agreement and values ranging from .61–.80 is substantial agreement (McHugh, 2012).

For example, students openly discussed their development of an identity as a “scientist” or “field biologist.” This construct is more formally recognized as science identity or the degree to which a student views themselves as the “type of person” to do science (Carlone & Johnson, 2007). Thus, to better understand students' identities within this field course, we integrated aspects of Gee’s (2000) Theory of Identity into our codebook so that we could consider whether and how students mentioned viewing themselves as scientists in their field journal reflections (Table 2).

Although the Model of the Affective Domain in the Geosciences (van der Hoeven Kraft et al., 2011), focuses on the motivational constructs of self‐efficacy, intrinsic motivation, and extrinsic motivation, we noticed that students discussed additional motivational components. Thus, to further understand students' motivational dispositions during biology field courses, we integrated Social Cognitive Theory (Table 2; Bandura, 1986) into our deductive coding approach. Through this process, we outlined components of motivation to learn science that we expected to see within student field journal reflections, in accordance with the science motivation model summarized by Glynn et al. (2011) (Table 2): extrinsic motivation (Mazlo et al., 2002; Ryan & Deci, 2000), intrinsic motivation (Ryan & Deci, 2000), self‐determination (Black & Deci, 2000), and self‐efficacy (Lawson et al., 2007). Due to the ecological focus of the course, we also included environmental motivation in our framework (Table 2) (Darner, 2009, 2012; Pelletier et al., 1998).

In their model, van der Hoeven Kraft et al. (2011) classify “connections with Earth” as the many ways humans value Earth, through aesthetic appreciation, emotional connection, or personal attachment to a specific location. We expanded upon this construct to become connection to nature, to better reflect the biological emphasis of our course and student language regarding connections they were making to organisms, ecosystems, and environments during field instruction (Table 2) (Restall & Conrad, 2015). In the context of the life sciences, this sentiment is also known as biophilia, a term first coined by Fromm (1973, pp. 365–366) and expanded upon by Wilson (1984) to describe an innate human tendency to care for all aspects of nature (Ulrich, 1993).

Lastly, to more holistically evaluate the emotions students experience during field instruction, we integrated Pekrun's Control‐Value Theory of Achievement Emotions (2006) to inform the deductive and inductive coding of emotions in student field journal reflections. This theory posits that students experience diverse sets of achievement emotions as they navigate educational settings and typifies the many emotions students may experience as they participate in the learning process. For example, activity‐focused emotions focus on feelings while engaged in an activity (e.g., going on a field trip) and outcome‐focused emotions focus on prospective or retrospective feelings about something that happened in the course (e.g., failing a test). These emotions can further be categorized by valence; that is, positive emotions are typically pleasant and imply a positive experience outcome and negative emotions are unpleasant and suggest negative experiences/outcomes (Pekrun et al., 2009; Plutchik, 2001).

On the fourth round of coding, the three coders reached consensus and thematic saturation, arriving at a final version of the codebook.

#### 3.2.2 Validity and reliability

Once we arrived at the final version of our codebook, we employed several methods to ensure validity, trustworthiness, and reliability in our qualitative analysis. Qualitative validation describes the measures researchers take to ensure their findings are accurate and representative of their experiences as well as the experiences of participants within the specific study context (Angen, 2000; Creswell, 2007). While there are several methods to establish validity, Creswell (2007) suggests that qualitative researchers undertake at least two validation procedures to ensure the trustworthiness of their findings.

We employed three validation procedures to verify that our findings were representative of the affective domain as it was expressed by students in the undergraduate field course. First, we provide a thick description, a textual representation intended to present a detailed picture of the participants and setting under investigation, of the undergraduate field course studied (see Section 3.1.1). A thick description allows the reader to discern whether the findings of a qualitative investigation apply and can be transferred to a different context (i.e., transferability) based on similarities or differences to the context described in the research (Lincoln & Guba, 1985).

For the second validation procedure, we used peer debriefing sessions, in which we met with and presented our codebook to two research groups composed of individuals with expertise in discipline‐based education research. Peer debriefing sessions are an exercise to establish credibility, wherein the researcher meets with others to obtain feedback on coding practices and ensure that the research findings are representative of participants' voices, meanings, and experiences (Lincoln & Guba, 1985). Based on the feedback we received during peer debriefing sessions, we revised several code descriptions to add clarity and to state them in a more neutral way to more equally capture both positive and negative affective sentiments present in our dataset (e.g., “a student *values or disvalues* a specific field experience…”, “a student describes independent actions they *will or will not* take…”). Additionally, based on peer feedback, we combined a small number of related and overlapping codes that helped to simplify our codebook.

Lastly, we included a positionality statement that describes the disciplinary background of all authors and indicates the relationship that any authors have with the course being studied (see Section 3.1.3). Positionality statements disclose experiences, perspectives, and biases that guided and could have influenced the research process (Lincoln & Guba, 1985; Merriam, 1988).

To statistically estimate the reliability of two coders' independent interpretations of our finalized codebook, authors D.E. and K.T. separately coded the same, randomly selected subset of previously unanalyzed field journal reflections (comprising 5% of the dataset). Following, we calculated Cohen's *κ* (Salkind, 2010) and percentage agreement, both of which measure the agreement between two coders to provide an indicator of the reliability of the coding results. The coders exhibited an overall high level of agreement (Cohen's *κ* = .896, 92.1% overall agreement). Cohen's *κ* values ranging from .810–1.000 are considered to be almost perfect agreement (McHugh, 2012). Cohen's *κ* values at the parent code level can be found in Table 2.

#### 3.2.3 Coding

Upon the completion of reliability calculations, two coders (D.E. and K.T.) split the dataset evenly into two halves and each coded one‐half of the complete dataset of field journal reflections using the finalized codebook and the software *nVivo 12 Plus* (QSR International Pty Ltd., 2018). The same two coders met regularly throughout this process, discussing and coming to a consensus decision about disagreements and coding segments where at least one researcher was unsure about the presence or absence of affect. In some cases, coding categories were inductively coded a second time by a single coder, with the goal of characterizing the breadth of the code and to identify common themes within.

#### 3.2.4 Analysis

Our goal for this paper was to characterize the affective responses that students discuss when reflecting on field experiences that are part of an introductory campus‐based field biology course. While our approach to this research allowed us to capture student affect broadly, our data and methods do not provide an appropriate quantitative measure of the affective constructs experienced by a single student. For instance, a student may have felt that one or many field labs made them feel like a scientist, but they may not have written about this affective disposition across each one of their journal entries. Apart from reporting unusually common or uncommon subcodes within our dataset, our study design is not suitable for quantifying the prevalence of affective responses; therefore, we largely deemphasized reporting quantities in this study. Instead, by leveraging the strengths of the wide variety of open‐ended responses elicited by our general prompt, we present a descriptive summary of each of the affective responses expressed by students in our dataset of 743 field journal reflections.

## 4 RESULTS

### 4.1 Framework of Student Affect in Field Biology

**FIGURE 3:**
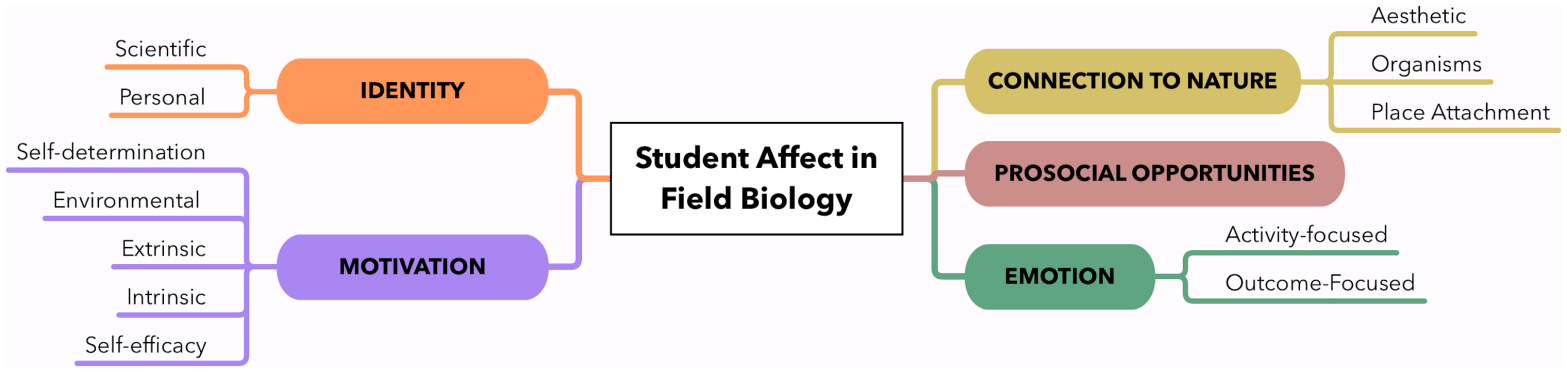
Framework of Student Affect in Field Biology

Informed by our analysis of field journal reflections, we propose the Framework of Student Affect in Field Biology, a five‐part framework describing the affective responses that students discuss when reflecting on field experiences in introductory field biology (Figure 3). This framework includes identity, motivation, connection to nature, prosocial opportunities, and emotion. In addition, this framework draws upon multiple social and psychological theories (see Section 3.2.1) to expand upon the Model for the Affective Domain in the Geosciences (van der Hoeven Kraft et al., 2011). Definitions for each of the constructs included in our framework are in Table 2, with each discussed in broader detail with accompanying exemplar quotes presented below.

### 4.2 Students reflected on their identity while in the field

We found that students discussed their identity through either a personal lens, scientific lens, or both (Table 3). Students described experiences that made them feel like field biologist (quote A, Table 3), that affirmed their intended major or career path (quote B, Table 3), and that reminded them of prior experiences (quotes C and D, Table 3). Notably, even within the first 2 weeks of field labs, multiple students mentioned viewing themselves as a “field biologist.” Students also described the way their fieldwork experiences related to who they are as people—what they like or dislike (quotes G and H, Table 3), where they come from (quotes I and J, Table 3), and how they grew up (quotes E and F, Table 3).

**TABLE 3:** Common “identity” themes within field reflections, with exemplar quotes

Theme	Quote	Exemplar quote
Feel Like a Field Biologist	A	“I felt like a true scientist completing valuable work”
Affirms Major or Career	B	“This reassured me I am studying the right thing”
Past Experience	C	“I’m not used to this kind of weather, and I’m also not used to going outside into the woods”
	D	“I have some experience in handling, and capturing fish, but I never really learned about the intricate roles all the different species play in the lake”
Growing Up	E	“I spent a lot of my childhood looking around streams and lakes”
	F	“Amphibians and reptiles have always interested me since when I was young and would play with newts in my backyard”
Likes/Dislikes	G	“I am very passionate about aquatic biology”
	H	“I have never been a bug person, I am scared of them, they freak me out, bugs have just never been my forte”
Where I am From	I	“As a southern boy, the first snowfall still brings me a childlike giddiness that brightens my spirit”
	J	“Being from Yonkers, the only wild birds I ever really see are pigeons, sparrows, crows, and geese”

Students varied widely in their prior field research experience (Figure 1b); however, only nine students indicated that they had moderate to no prior outdoor experience (Figure 1a). Some of these students referenced this lack of experience when describing first‐time research experiences in class. One student wrote: “This lab was the first time we worked mainly on our own without direct help from instructors. For this reason, I found it to be engaging and challenging at points because I had never done anything like this before.” Another student referenced their insecurities about collecting field data as a novice: “I am sure that the procedures we were using make sense to experienced foresters, or people who have been given proper training, but for us the instructions were hard to interpret, and our results felt more like guesses than accurate data collection.”

### 4.3 Students described various motivations for participating in field labs

Within field journal reflections, students described multiple motivations for engaging with field labs (Table 4). Intrinsic motivation (Ryan & Deci, 2000), was by far the most common orientation of motivation discussed in field journals, with, on average, half of the students referencing intrinsic motivation within each reflective journal entry (quotes A and B, Table 4). Despite this frequency, students often failed to elaborate further on the nature of their *i*ntrinsic motivation, opting for vague terminology such as “cool,” “interesting,” or “awesome” but neglecting to say *why* they expressed these value statements.

**TABLE 4:** Common “motivation” themes within field reflections, with exemplar quotes

Theme	Quote	Exemplar quote
Intrinsic motivation	A	“This field trip ultimately re‐iterated to me why I am taking the course—to explore the natural wonders of the world around us”
	B	“It made me curious about the evolutionary cause for the development”
Extrinsic motivation	C	“I think this trip really reaffirmed for me that I want to work in a field setting one day and gave me some ideas for what types of internships I may want to apply to in the future”
	D	“This lab was very interesting in that I could see myself carrying out activities like this as a career”
Environmental motivation	E	“… studying insects is crucial to understanding ecosystem sustainability so I appreciate the exposure”
	F	“I feel like this is a very important and applicable topic to many issues we are having in the natural world today”
Self‐determination	G	“From this activity I realized that I need to work more on my binocular skills because often I would be able to see the bird with my eye but be unable to find it in my binoculars quickly enough and it would already have flown away before I was able to identify the correct branch. I think that I will be able to overcome this challenge with more practice”
Self‐efficacy	H	“I also learned that I am not great at telling birds apart on the fly, so that is definitely a skill that I need to actively work on”
	I	“… after going on tree walks, learning about TAs [teaching assistants] personal experiences, and having nothing but tree ID on my mind for 36 hours, I became more confident in my ability to ID trees. It is kind of like a superpower now”

Additionally, students expressed sentiments related to their science self‐efficacy when performing content‐related tasks during the field labs. Students noted when they felt more confident in their abilities to complete protocols, identify species (quote I, Table 4), and participate in fieldwork. Students also recorded situations when they lacked science self‐efficacy, for example, when they felt unsure of their abilities to perform certain content‐related tasks (quote H, Table 4). In these cases, students often described that they would undertake efforts to improve in these areas.

Students also described their extrinsic motivation (quotes C and D, Table 4) (Ryan & Deci, 2000) for engaging in field labs. Notably, we found students were far more likely to discuss being motivated by career goals or a desire to pursue future avenues of academic interest rather than being motivated specifically by course grades.

Another motivational construct discussed in the field journal reflections is environmental motivation (quotes E and F, Table 4) (Darner, 2009; Pelletier et al., 1998). Students described how a desire to protect and care about nature motivated them to participate in certain field activities such as identifying local species, collecting data about ecosystem health, or visiting field sites that have been impacted by human activity.

While uncommon in students' field journal reflections, we found evidence for students exhibiting self‐determination, or desire to engage in self‐regulated learning outside of the field course (quote G, Table 4). Self‐determination was mostly exhibited when students experienced failure (e.g., when failing an exam and feeling the need to study the course material outside of class time) or expressed interest in exploring the topics covered in the course in their free time (e.g., a student committed to reviewing avian field guides to get better at bird watching after participating in the ornithology unit).

### 4.4 Students formed deep connections with the nature around them

In field journal reflections, students described connecting to nature through appreciating the beauty of natural places, organisms, and weather (Connection to Aesthetic) (quotes A and B, Table 5), connecting to organisms they interacted with in the field (Connection to Organisms) (quotes C and D, Table 5), and forming attachments to specific locations they visited (Place Attachment) (quotes A, E, F, Table 5). Students formed connections to various field sites through appreciating their aesthetic qualities, experiencing positive emotions, and valuing qualities about certain sites that made them particularly well‐suited for the study of field biology and connecting with nature.

**TABLE 5:** Common “connection to nature” themes within field reflections, with exemplar quotes

Theme	Quote	Exemplar quote
Connection to Aesthetic	A	“It was a very beautiful location and I loved the trails”
	B	“I thought it was so incredible to see a Blue Jay up close, as I think they are a really beautiful species of bird”
Connection to Organisms	C	“… I had never looked closely at zooplankton before and I never really understood their role in the lake ecosystem. I thought it was fascinating to get a close up look at these nearly invisible organisms that are so vital to all life in a lake”
	D	“One thing that I thought was pretty cool was that we found a cucumber tree, Magnolia acuminata. I had never heard of this tree before and thought it was super odd…. I thought the leaves on this tree were really cool…”
Place Attachment	E	“I loved the location for this lab”
	F	“I was so excited about the place that I almost convinced my mom to make a trip out there with me that weekend to visit”

**FIGURE 4:**
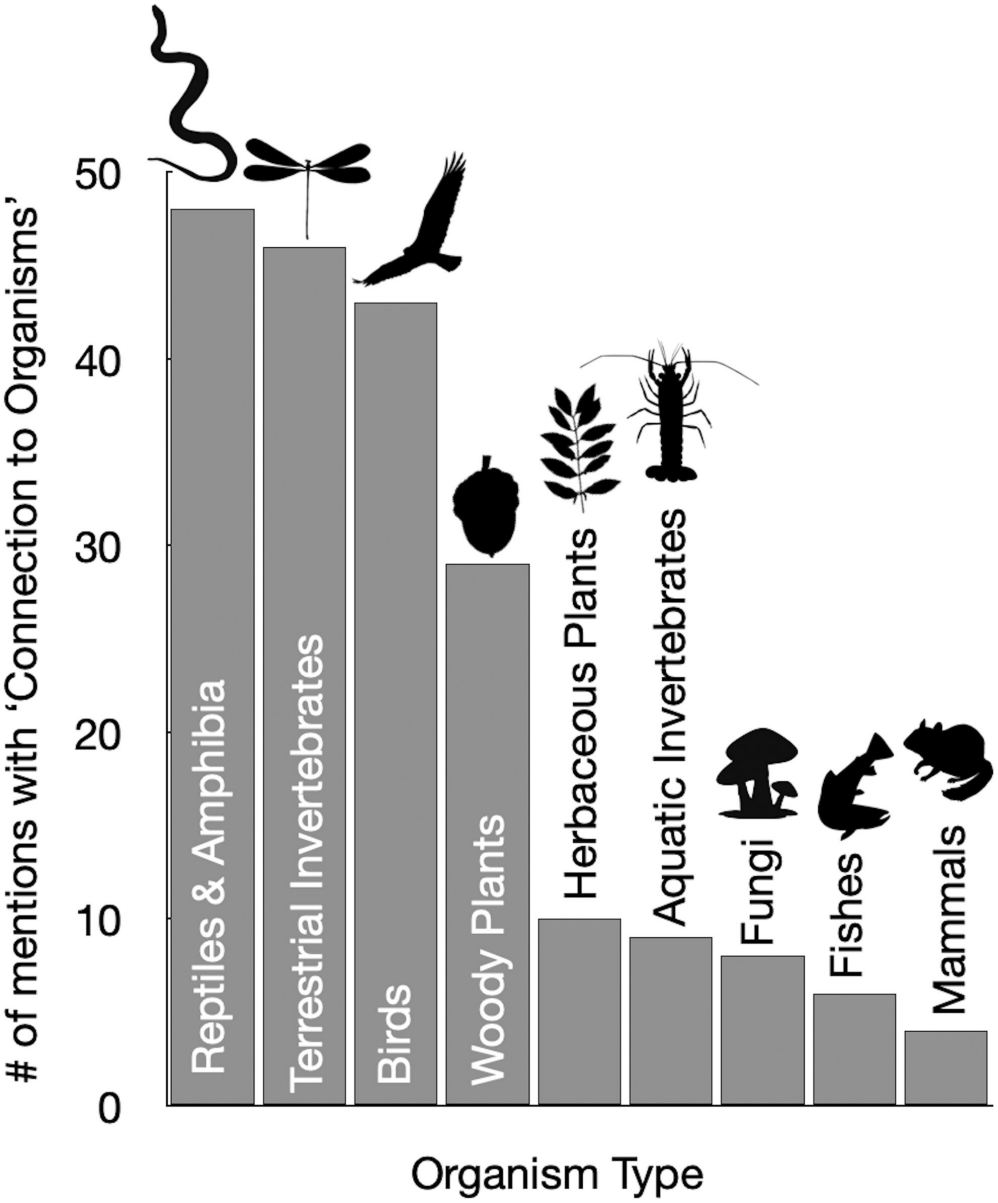
Frequency of organism groups mentioned in field journal reflections

Students also described meaningful connections that they made to living organisms they physically interacted with during field labs, including reptiles and amphibians, terrestrial invertebrates, birds, trees, and other taxa (Figure 4). Students frequently described making connections to these organisms through the act of discovering, most commonly through finding or catching, but also from seeing a specific organism for the first time or identifying an organism themselves. Students used a variety of senses while engaging with organisms, including visually observing, smelling, hearing, or touching (quote C and D, Table 5). Notably, students often reflected on their encounters with organisms by detailing an emotional response they felt towards organisms, such as fear, excitement, appreciation, or affection.

Students with less prior outdoor experience (Figure 1a) sometimes emphasized how their lack of outdoor experience contributed positively to their novel interactions with organisms. One student wrote: “This lab exercise was interesting because prior to this activity I never had any experience with identifying birds at all. My knowledge of birds was really limited to pigeons and in New York City, we treat them like first class citizens, not birds. But this experience really gave me a whole new set of information regarding birds! Now I’m able to identify a turkey vulture based on the way it flies (teetering and in a V shape), as well as chickadees and catbirds based on their calls, and barred owls too.”

### 4.5 Students expressed positive attitudes towards social interaction

In field journal reflections, students wrote about interactions they shared with the individuals they socialized with during their field labs including classmates, undergraduate, and graduate teaching assistants, professors, and visiting lecturers (Table 6).

**TABLE 6:** Common “prosocial opportunities” themes within field reflections, with exemplar quotes

Theme	Quote	Exemplar quote
Learning from TAs [teaching assistants], Instructors, and Visiting Lecturers	A	“I also thought it was really helpful to go on a tree walk with the UTAs [undergraduate teaching assistants]. They had tricks to memorize species that I had never heard of before and nice tips for taking the tree exam in general. Also, it was nice to talk to people who had already taken tree exams as they knew exactly what we were going through”
	B	“She also provided us with some helpful hints for the future. She explained how her career path didn’t go exactly as planned, and yet she is incredibly happy and loves what she does. It is certainly a valuable lesson that things in life don’t always go the way you expect, but sometimes it’s for the best”
Positive Group Attitudes	C	“Today was one of the rare times that working as a group was not only easy, but actually enjoyable”
Negative Group Attitudes	D	“Since the groups were relatively large, with about five people per group it was difficult to come to a consensus. I found that we spend a great deal of time trying to compromise on what we should put as the value for certain metrics instead of thoroughly analyzing the health of the tree”
Interpersonal Connection	E	“I also think [this field trip] brought us closer as a class which isn’t the case in a lot of the classes I am in, so this one feels really special. We all really bonded and it was an amazing and valuable experience”
	F	“Overall, I felt that this trip allowed me to get to know my professors, TAs and fellow students better while having a true immersive experience in the subject material”
Explaining Science	G	“Even after only one day of class, when I walked around campus and through the botanic gardens during the rest of the week. I was able to recognize a few species like the Tower Rd. red oaks and point them out to my friends”

Students were positive about the social opportunities afforded by field instruction (quotes A–C, E–G, Table 6). Further, students described interactions they had with instructors and undergraduate TAs (quote A, Table 6), which they found encouraging, reaffirming, and helpful for their understanding of the course content. Students also appreciated the opportunity to learn content and obtain career insights from visiting lecturers (quote B, Table 6). Students were similarly positive about interactions with their peers, appreciating how cooperative learning opportunities (i.e., group work) during field labs helped them form social relationships, which were often helpful for the completion of the course assignments (quote C, E, F, Table 6). However, one student complained about the difficulties of reaching a consensus on experimental decisions when working in a group (quote D, Table 6). Students also mentioned sharing and explaining the knowledge accrued during the course to nonclassmate friends and family. For instance, students expressed enthusiasm about their ability to identify and share their knowledge about organisms with their friends (quote G, Table 6). One student described an instance where they sent their mother pictures of what they accomplished after a day of field activity.

### 4.6 Students felt a wide array of emotions in the field

We found that students regularly expressed a wide array of activity‐focused or outcome‐focused achievement emotions in their field journal reflections (Tables 7 and 8).

**TABLE 7:** Emotions expressed in student's field reflections

Type of emotion	Emotion	# Of coded segments
Activity‐Focused	Enjoyment (+)	625
	Relaxed/Calm (+)^a^	18
	Surprise (+/−)^a^	99
	Frustration (−)	7
	Anger (−)	0
	Boredom (−)	7
Outcome (Prospective)	Anticipatory Joy (+)	30
	Hope (+)	1
	Hopelessness (−)	1
	Anticipatory relief (−)	14
	Fear/Anxiety (−)	60
Outcome (Retrospective)	Joy about success (+)	12
	Pride (+)	16
	Satisfaction (+)^a^	13
	Gratitude (+)	8
	Sadness (−)	25
	Shame/Guilt (−)	10
	Dislike/Disappointment (−)^a^	35
	Anger (−)	0

^a^ These emotions were inductively coded and integrated into the Control—Value Theory of Achievement Emotions (Pekrun, 2006) Framework. Emotional valence is indicated in parentheses, where + represents a positive emotion, − represents a negative emotion, and (+/−) represents a neutral emotion.

**TABLE 8:** Common “emotion” themes within field reflections, with exemplar quotes

Theme	Quote	Exemplar quote
Activity‐focused emotions	A	*Enjoyment*: “Being someone who learns better with engaging, hands‐on learning, I really enjoyed this lab activity because it allowed me to actually use the methods I had learned prior to this lab section in a real‐life setting”
	B	*Surprise*: “I definitely was surprised again with how easily I was able to jump into a foreign activity. During this lab, I caught just as many insects as some of my more experienced classmates, and even mustered up the courage and caught a wasp without getting stung (and thankfully no one else got stung either)”
Outcome‐focused emotions (Prospective)	C	*Fear/Anxiety*: “I was a bit worried about the lab. Being essentially in the middle of the forest to do this lab made me wonder how I got up to this point in my life; however, I still had a job to do”
	D	*Anticipatory Joy*: “It makes me excited because I feel like by the end of the semester, I will be just as knowledgeable as [the undergraduate TA.]”
Outcome‐focused emotions (Retrospective)	E	*Dislike/Disappointment*: “Field bio is significantly less fun when the weather drops 30 °F in a week and you are outside learning about soil while it is snowing, but it was very pretty”
	F	*Sadness*: “Within the next 50 years or so, we are looking at major changes to these aquatic ecosystems and it’s sad to see that this is happening”

Enjoyment was the most prevalent emotion present in student field journal reflections followed by surprise (Table 7 and quotes A and B, Table 8). Students expressed enjoyment in many facets of field instruction such as fieldwork, learning new experimental procedures, and opportunities for social interaction with peers. While students mainly reflected on emotions elicited by field course activities and assignments,some wrote about their anticipatory feelings about events that happened or were going to occur in the field course (quotes C and D, Table 8). A subset of these segments focused on students' positive expectations of things to come in the course (e.g., looking forward to future research opportunities). However, the most frequently coded prospective outcome‐focused achievement emotion was fear and anxiety (Table 7 and quote C, Table 8). Some students described feeling fear and anxiety when interacting with terrestrial arthropods (e.g., wasps, spiders), taking exams, practicing new field methods, and learning about worsening global environmental conditions (Table 8). In particular, students who entered the class with less outdoor experience (Figure 1a), tended to reference their lack of experience alongside prospective outcome emotions of both anxiety and anticipatory joy. One student wrote: “This class is definitely going to be a challenge that I am excited to take on. I have nearly no experience being outdoors and actually observing natural surroundings, so I am ready to finally experience this aspect of living on a campus surrounded by nature. Hearing that we will have to memorize a few dozen species of plants did make me slightly nervous, because just from seeing the 12 species in today's tree walk, I feel my brain working very hard to try to remember the details that make identifying easier.”

Finally, students described feeling a variety of retrospective outcome‐focused achievements as they reflected on their successes and failures during field labs. The most common of these emotions expressed were disappointment and dislike of certain course activities such as culling invertebrate specimens and inclement weather conditions (quotes E and F, Table 8).

## 5 DISCUSSION

Field courses provide students with hands‐on experiences to learn by connecting theory and practice while immersed in the natural world. They are associated with increased student success and the promotion of certain positive affective outcomes (Beltran et al., 2020; O’Connell et al., 2021, 2022). Yet, they face logistical and support challenges that threaten their future within undergraduate education (Fleischner et al., 2017). To ensure the continued support of field courses, recent calls have sought a better understanding of their affective outcomes (Geraghty Ward et al., 2021; Jolley et al., 2018; National Research Council, 2012).

This study answers these calls by broadening our understanding of the affective outcomes students describe experiencing while participating in field biology courses. Using over 700 field journal reflections, we propose a new Framework of Student Affect in Field Biology (Figure 3, Table 2). Many of these constructs—such as motivation, self‐efficacy, and place attachment—are important for persistence and retention in STEM (Dou et al., 2016; National Research Council, 2012; Semken & Freeman, 2008). A subset of our findings is supported by prior studies on student affect in field courses, which explored individual affective outcomes such as science self‐efficacy (Race et al., 2021), science identity (Beltran et al., 2020), motivation (Scott et al., 2019), and prosocial attitudes towards peers and instructors (Peacock et al., 2018). In addition, we found evidence of students exhibiting a greater connection to the natural world, a finding observed in geoscience fieldwork experiences (e.g., Jolley et al., 2018) yet only now documented in the present study of life sciences field courses. Student field journal reflections also detailed a broader range of positive (e.g., enjoyment) and negative (e.g., anxiety) achievement emotions than is documented in the literature, both during and following their participation in biology fieldwork.

### 5.1 Using the Framework of Student Affect in Field Biology to encourage and assess affective outcomes

We encourage instructors to use the Framework of Student Affect in Field Biology (Figure 3) to describe intended affective learning outcomes goals for field courses (e.g., “As a result of participating in course field labs, students will develop stronger confidence in their abilities to do science”), and to explicitly list them alongside more traditionally enacted intended cognitive learning outcomes in course syllabi. Historically, biology education has focused on assessing the cognitive domain (Shinbrot et al., 2022; Trujillo & Tanner, 2014). However, following a backwards design process (Wiggins & McTighe, 2005), instructors can assess intended affective outcomes with journaling prompts or survey instruments with valid evidence (see Shortlidge et al., 2021 for review), and can design activities and assignments that are in alignment with assessments and affective outcome goals. To aid instructors during the backwards design process, we use the Framework of Student Affect in Field Biology to suggest journal reflection prompts that are targeted to explore and assess affective development in students (summarized in Table 9).

**TABLE 9:** Reflective journal prompts for exploring affective constructs in field biology courses

Construct	Targeted reflective journal prompt
Identity	“To what extent do you see yourself as a field biology person?” (modified from Hazari et al., 2013) “Did participating in this field experience change the way you see yourself as a scientist? If so, how?” “How do your past experiences shape the way you approach learning in the field?” “How did your outdoor and/or research experience prior to this course impact how you learned and participated in today's field experience?”
Motivation	*Motivation (broad)*: “How motivated did you feel during this field experience? Why?” (modified from Scott et al., 2019) *Intrinsic Motivation*: “What aspects of this experience did you find to be the most interesting or curiosity‐provoking? Why?” *Self‐efficacy*: “Did you do anything during this field experience for the first time? What was it like? How do you feel now after the experience?” (modified from Race et al., 2021) *Environmental Motivation*: “How does understanding this topic/learning this fieldwork skill help the environment?” (modified from Pelletier et al., 1998) *Extrinsic Motivation*: “How might this fieldwork skill/topic/experience help you to accomplish your academic or career interests?”
Connection to nature	“Did participating in this field experience change the way you feel connected to or disconnected from the natural world? If so, what aspect of this experience, and how did it change your feelings of connectedness?” “Describe any thoughts and feelings you have about: (A) the place where this field experience was held and (B) the organisms that you interacted with during this field experience”
Prosocial opportunities	(from Gaudet et al., 2010) “What is positive or beneficial about working in a group? “What is negative or challenging about working in a group?”
Emotion	(Modified from Boyle et al., 2003, 2007) “What are you feeling prior to participating in this field experience? Why?” “How do you feel after participating in this field experience? What aspects made you feel this way?”

### 5.2 Field labs contribute to science identity formation

Experiential learning in the field can serve to affirm existing identities or contribute to the strengthening or formation of new identities (Morales et al., 2020; Posselt, 2020; Posselt & Nuñez, 2022). We found that throughout the semester, students discussed their personal and scientific identity development after participating in field labs (Table 3). The results in this study are consistent with research that shows that when students reflect on their scientific identity, they will similarly reflect on aspects of their personal identities and prior experiences (Le et al., 2019).

A student's identity can influence actions, perceptions, and experiences, and thus can have a powerful influence on learning outcomes (Bell et al., 2018; Brickhouse et al., 2000; Gee, 2000; Henning et al., 2019; O'Connell et al., 2022). Particularly salient for educators, increased scientific identity has been correlated with increased motivation to persist in STEM, educational success, and a sense of belonging (Kang et al., 2018; McDonald et al., 2019; Stets et al., 2017). Notably, science identity plays an important role in supporting student success in science, particularly for those who have traditionally been excluded from STEM disciplines (Carlone & Johnson, 2007; Estrada et al., 2016; Hernandez et al., 2013; O'Connell et al., 2022). Therefore, a better understanding of the interaction between fieldwork experiences and students' scientific and personal identities is an important step instructor can take towards building a supportive and inclusive learning environment for all students. As identity is socially and contextually constructed, engaging in immersive field labs may impact how a student sees themselves as a field biologist, connects to the environment, or connects to others within outdoor spaces (Clayton & Opotow, 2003; Hughes, 2016; Seymour et al., 2004; Streule & Craig, 2016; Williams & George‐Jackson, 2014).

If increased science identity is a desired affective outcome, instructors can learn more about student identity using reflection prompts (Table 9) to learn about the many facets of identities that are at play when students participate in field labs. We recommend that instructors take these many identities into account when designing, preparing for, and leading inclusive field courses, for example, knowing that certain students grew up with limited prior outdoors and/or field research experience. An instructor might choose to incorporate anonymous opportunities for students to pose questions that can be addressed prior to going into the field.

### 5.3 Students have a variety of motivations to participate in field courses

Motivation mediates self‐regulated learning by harnessing interest and engagement on a topic, which can lead to more successful learning outcomes such as deeper engagement, greater persistence, and higher performance (National Research Council, 2012). The engaging and immersive nature of field courses increases student motivation by focusing students' attention (Ballantyne et al., 2010) and providing opportunities for independent and self‐directed learning (Scott et al., 2019). We found that students' field journal reflections detailed frequent instances of motivation (Table 4), in particular, intrinsic motivation, which was prompted by unique opportunities this course provided for immersion in natural areas on and near campus and to pursue their interests about the natural world.

Students noted the ways field labs inspired them to want to pursue similar future opportunities in field biology (e.g., undergraduate research positions, internships) when writing about extrinsic motivation factors. Previous research has shown that external motivation is likely mediated by both positive self‐efficacy and science identity (Chemers et al., 2011)—two other themes present in students' field journal reflections (Tables 3 and 4). Pertinently, a previous comparison between field‐based and classroom‐based tasks indicates that students found field‐based tasks to be more valuable to their attainment of skills related to their career goals (Scott et al., 2012). Similarly, field courses have been shown to be associated with academic success, including higher graduation rates, retention in majors, higher graduation GPAs, and gains in self‐efficacy (Beltran et al., 2020; Kortz et al., 2020).

We also found that students valued field learning for its alignment with their desire to learn about and appreciate the natural world (Table 4)—a concept known as environmental motivation (Table 2). Environmental motivation, an implicit goal of many environmental education programs, has been associated with an increased sense of belonging and desire to care for the environment, a desire to learn about the environment, and has been correlated to pro‐environmental behaviors (Bramston et al., 2011; Darner, 2009, 2012; Pelletier et al., 1998).

Understanding the breadth of motivational orientations among students in field courses can help instructors design field exercises that are inviting, accessible, and useful to all students (Scott et al., 2019). Instructors wanting to learn more about student motivation can ask for targeted motivational prompts (Table 9). Targeted prompts may help reduce students' use of vague descriptions such as “cool” or “awesome” in favor of eliciting deeper and more thorough reflections. In addition to providing instructors with valuable information, deeper reflection on motivation may also support students in the discovery and pursuit of opportunities that better align with their interests.

### 5.4 Students benefit from opportunities to connect with nature

We found that students described connecting to nature through appreciating its beauty, by forming attachments to various field sites and connecting to organisms they interacted within the field (Tables 2 and 5). Connections to the natural environment like these have been shown to strengthen students' attachment to place, increase value, and aid in development of ecological identity, including nurturing a desire to care for and motivation to learn about the natural world (Boyle et al., 2007; Jolley et al., 2018; Semken & Freeman, 2008; van der Hoeven Kraft et al., 2011). Furthermore, pedagogy that fosters students' connection to place has been shown to increase engagement and retention, particularly for minoritized students from indigenous communities, which often possess centuries‐old systems of place‐based ecological knowledge (Gibson & Puniwai, 2006; Kawagley et al., 1998; in Semken & Freeman, 2008).

Place attachment is associated with positive emotional and social outcomes and increased wellbeing, and has also been shown to be significantly correlated with pro‐environmental behaviors. Thus, to facilitate students' place attachment, instructors can provide multiple experiences connected to the same place over time (Kudryavtsev et al., 2012). For instance, field course instructors may ask students to visit one or a few field sites and document changes in the ecology of the area over the course of the semester.

We also found that students formed connections to nature through interacting with organisms in the field (Figure 4 and Table 5). While observation is one well‐established means of eliciting connections to organisms and a long‐standing learning goal of many field courses (Fleischner et al., 2017; Mogk & Goodwin, 2012), students also described making connections to organisms through self‐discovery. Notably, prior research has found that course‐based undergraduate research experiences that scaffold opportunities for students to engage in scientific discovery can enhance the degree to which students engage with and feel ownership over their work (Cooper et al., 2019). As such, we encourage field course instructors to incorporate more opportunities for self‐discovery into field courses, for example by setting aside course time for students to search for and identify organisms at field sites, providing specific advice on best practices for locating and identifying organisms in the environment, and encouraging opportunities for students to share their discoveries with their peers.

In addition to fostering positive affective benefits, increased connections to nature can improve student mental health (Mayer et al., 2009). Field courses present particularly valuable opportunities to nurture students' nature connectedness. These benefits are particularly valuable in the face of growing mental health challenges that students face on college campuses in the U.S. (Posselt & Lipson, 2016). Contemplative pedagogical practices (e.g., silent sitting, deep listening) have been shown to decrease students' stress, improve their sense of wellbeing, increase their focus, and support deeper learning (Faerm, 2020; Zajonc & Sanders, 2013). Targeted reflection prompts (Table 9) can guide students to reflect more deeply on their connection to nature and provide instructors with a means of determining how a student's connection to nature may have changed over time.

### 5.5 Field courses provide unique opportunities for social interaction

Overall, we found that students valued the potential for social interaction in the field course for its ability to help them learn about the life sciences and build relationships with their peers, instructors, and others (Table 6). These findings are well aligned with “Prosocial Opportunities” of the Model of the Affective Domain for the Geosciences (van der Hoeven Kraft et al., 2011). While social relationships have been relatively understudied in the context of field biology education (Mason et al., 2018), evidence from geoscience education research suggests that field courses can help promote the formation of social relationships among students and between students and instructors (Boyle et al., 2003; Jolley et al., 2018; Petcovic et al., 2020; Streule & Craig, 2016). We found evidence for similar types of social engagement and relationships in the present study, in addition to new aspects of social interaction previously undocumented in field courses (e.g., explaining science to others outside of the course). Students frequently described positive connections they made with peers, which is concurrent with the results of Peacock et al. (2018), who highlight the potential for undergraduate field courses to promote both formal interactions centered on student learning and informal interactions that foster interpersonal connection among students.

Group‐based learning is a common feature of field courses for logistical, pedagogical, and safety reasons (Scott et al., 2019; Streule & Craig, 2016; van der Hoeven Kraft et al., 2011). As such, field course instructors can play an important role in fostering positive social interactions among students, thus helping them gain valuable teamwork skills and capitalize on the cognitive and affective benefits of cooperative learning (van der Hoeven Kraft et al., 2011). Furthermore, studies indicate that the number of interactions as well as students who form social relationships with is predictive of their self‐efficacy and persistence in their major (Dou et al., 2016; Zwolak et al., 2017). Similarly, students who exhibit higher interactivity with peers and instructors in biology laboratory courses show developments in a variety of motivational orientations, science identity, and science self‐efficacy (Esparza et al., 2020).

Given these benefits, instructors can support prosocial interactions by making thoughtful decisions about how groups are formed (Donovan et al., 2018), and how roles within groups are fairly allocated, or by incorporating opportunities for long‐term, sustained group work in the form of a collaborative research project (Auchincloss et al., 2014; Heller & Hollabaugh, 1992). If developing positive attitudes towards teamwork is an intended affective outcome of the course, instructors can assess students using targeted reflective writing prompts (Table 9).

### 5.6 The importance of considering students' emotions during field courses

Several studies indicate that students feel positive emotions such as enjoyment (Kern & Carpenter, 1984; Scott et al., 2019; Van Loon, 2019) during field‐based instruction. However, other research has found that students can also express negative emotions during field instruction such as frustration (Baum et al., 2012), boredom (Goulder et al., 2013; Orion & Hofstein, 1994), and even fear (Brenner et al., 2018).

Overall, we found that students predominantly described their enjoyment of various aspects of the undergraduate field course when writing their field journal reflections (Table 7). However, students also displayed negative activity and outcome‐focused achievement emotions in their field journal reflections (Table 8). Research indicates that students' achievement emotions are tightly associated with academic performance, motivation, self‐efficacy, and feelings of belonging (Lam et al., 2015; Pekrun et al., 2011). As such, it is imperative to consider what types of field activities elicit positive emotions in students and which may cause students to feel negative emotions.

One way to mitigate students' negative emotions may first be to provide them with a detailed description of what to expect from each field excursion, including the amount of physical labor required, the amount of time that will be spent in the field, certain types of equipment to bring, appropriate clothing, and the specific field practices to be undertaken (Butler, 2008). Importantly, these practices have been suggested to be particularly helpful in supporting neurodiverse students (Kingsbury et al., 2020). Involving students in these pretrip briefings may allay some of the students' anxieties, apprehensions, and fears (Boyle et al., 2007). Alternatively, negative emotions could be mitigated by providing students with alternative options to fieldwork activities that they fear or dislike (e.g., allowing students to release insects and study existing preserved specimens rather than culling specimens they have collected).

Still, it is likely that negative emotions will occur during field instruction, for example in response to unexpected events, such as chance encounters with unfamiliar organisms or due to misaligned expectations of the fieldwork activities. In this sense, we recommend that instructors engage their students in reflective journaling to better capture students' achievement emotions as they engage in fieldwork (Table 9).

### 5.7 Study limitations

Field journal reflections provide a unique window into student affect by capturing students' impressions of memorable aspects of field labs. These reflections can help students process and learn from their experiences (Boud et al., 1985; Kolb, 2015) and, likewise, benefit instructors by providing direct feedback about the possible outcomes of individual field labs (O’Connell & Dyment, 2011; Tammu, 2022). When compared to reflections elicited by specific outcome‐oriented prompts, general prompted reflections are less bounded and may capture a broader assortment of responses. Moreover, reflective journaling is low cost and more time‐efficient to collect and review as compared to ethnographic observations and semi‐structured student interviews (Hodder, 2000).

Despite their pedagogical and research utility, field journal reflections are limited by *how* students describe their affect and experiences in the field course. While field journal reflections capture rich qualitative data, they are bound by language, which may not accurately portray the overall complexity of students' experiences and affective dispositions (Suzuki et al., 2007). Here, we found evidence of a diverse array of affective responses. Still, it is possible that undergraduate field courses can elicit additional types of affective responses despite their absence in our dataset. For this reason, we view field journal reflections as a valuable but likely incomplete assessment of student affect. We recommend the use of prompts (Table 9) alongside other assessments (see Shortlidge et al., 2021 for a review of assessment tools) to understand student outcomes.

Further, field journal reflections are limited by *what* students decide to write. Reflective journaling can cue emotions and experiences that may be sensitive for students to remember and reflect upon (Ghaye, 2007). As a result, a student may not feel comfortable or choose to omit certain affective responses to their field lab. Moreover, it is possible that the nonanonymous nature of the journal assignment may have resulted in social desirability bias. Social desirability bias can occur when students provide responses they perceive to be desirable to instructors in an effort to get a better grade, rather than respond with their true beliefs, attitudes, or values (Paulhus, 1991). This phenomenon is known to occur when collecting reflective field journal responses from students. In their review of the literature, O’Connell and Dyment (2011) discuss the potential for students to “write for the instructor,” a process by which students may censor their field journal reflections in certain ways in an effort to gain full credit for the assignment. For instance, students may only write about what they expect their instructors want to read, such as aspects of the course they enjoyed, or omit negative response for fear of grade repercussions (Crème, 2005; Sutton et al., 2007). In this study, we graded students' reflective journal entries for completion rather than content to support robust reflective practice and a course learning outcome of writing a thorough and detailed field journal. Still, it is possible that social desirability bias may at least partially explain why our dataset predominantly includes positive affective reflections. It is also possible that students may feel inhibited or unaccustomed to sharing personal details while journaling, an observation recorded by Sutton et al. (2007). Particularly in STEM courses—where scientific research is often emphasized to be objective and impersonal—students may be unfamiliar with or uncomfortable writing about aspects of their personal identity, emotions, or values.

Other limitations of the present work include the generalizability of the results to other field course contexts. The data analyzed in this research represent field journal reflections from students in a campus‐based field biology course who tended to have prior outdoors experience (Figure 1a). Undergraduate field courses can be taken by different student populations and delivered in many modalities (e.g., residential field courses, field camps). While research on other student populations and field course modalities has revealed similar outcomes to the present research (Race et al., 2021; Scott et al., 2019), it is possible that students would display other affective responses in another type of fieldwork experience. Lastly, the field course we studied included learning outcomes that were focused on supporting student social interaction, science self‐efficacy, and science identity. Therefore, it is possible that students could have achieved greater affective growth through participating in the field course and were, therefore, more likely to write about their interests, motivations, and beliefs.

### 5.8 Next steps

Field journal reflections have great potential for helping instructors and researchers better understand the way students experience field courses, and thus improve field‐based education. When collected regularly throughout a course, the field journal reflections comprise a high‐resolution temporal dataset that enables researchers to directly link affect to experience. For instance, future research can investigate how different field labs throughout a semester impact students' affective development. Drawing on the Framework of Student Affect in Field Biology (Figure 3), future studies should compare the way students link affect to experience in field journal reflections from different field course types and at different institutions.

Lastly, beyond providing a window into student affect, field journal reflections included unique cognitive insights into the way field labs helped students understand, appreciate, and reflect on the scientific process. In field journal reflections, students also expressed curiosity about biology, generated hypotheses about the natural world they observed, discussed their perceptions about the scientific research process, and shared their opinions about their confidence in the data they collected. Students shared what aspects of the course they enjoyed as well as what challenges they encountered, both of which offer invaluable feedback for field course instructors wishing to iteratively improve upon course materials for the future. Given the affective outcomes students described when leaving the classroom to learn outdoors, we see great potential for incorporating fieldwork opportunities throughout undergraduate biology curricula.

## AUTHOR CONTRIBUTIONS

**Kira A. Treibergs:** Conceptualization (lead); data curation (lead); formal analysis (equal); investigation (lead); methodology (equal); project administration (lead); supervision (lead); validation (equal); visualization (lead); writing – original draft (equal); writing – review and editing (equal). **David Esparza:** Conceptualization (supporting); formal analysis (lead); investigation (lead); methodology (lead); project administration (equal); validation (equal); visualization (equal); writing – original draft (equal); writing – review and editing (equal). **Jeannie A. Yamazaki:** Investigation (supporting); validation (equal); writing – review and editing (supporting). **Marc Goebel:** Methodology (supporting); validation (supporting); writing – review and editing (supporting). **Michelle K. Smith:** Conceptualization (supporting); funding acquisition (lead); project administration (equal); supervision (supporting); writing – review and editing (equal).

## CONFLICT OF INTEREST

The authors declare that the research was conducted in the absence of any relationships or interests (commercial, financial, or otherwise) that could be construed as a potential conflict of interest.

## Data Availability

The datasets referenced in this article are not readily available because the approved study protocol and consent form explicitly state that data will not be shared with external parties except in instances where the research team is required to do so by law. Requests to access the datasets should be directed to the corresponding author Kira A. Treibergs, Ph.D. (kt596@cornell.edu).
